# Supplementary material for: The geometry of meaning: evaluating sentence embeddings from diverse transformer-based models for natural language inference
Source: PeerJ Comput Sci. 2025 Jun 16;11:e2957. doi: 10.7717/peerj-cs.2957 (PMC12193426; doi:10.7717/peerj-cs.2957)
Supplement: Supplemental Information 1 [file peerj-cs-11-2957-s001.pdf]

1. Introduction

Motivation for the Study:

This study aims to optimize transformer-based models for Natural Language Inference (NLI), a key task in natural language processing. NLI requires understanding the relationship between sentence pairs, making it crucial to generate accurate sentence embeddings. By systematically exploring different pooling strategies and norms across transformer models, this work seeks to provide insights into improving model interpretability and sentence-level representation. Additionally, we extend the original binary entailment framework to support full three-way classification, and evaluate performance across both general and domain-specific datasets.

2. Reproducibility

Code Submission:

The code is attached as a supplemental file (geometry.py) and is also available at the following GitHub repository:

https://github.com/suhaibani/geometry

README file:

The readme file is attached as a supplemental file (README.md) and is also hosted in the repository above.

3. Materials and Method

Computing Infrastructure:

All experiments were conducted on a system running Ubuntu 20.04 with an NVIDIA Tesla T4 GPU. The models were implemented using Python and PyTorch, with the Hugging Face Transformers library used to facilitate model loading and sentence embedding.

3rd Party Datasets:

We evaluated our approach on two public datasets:

- SNLI (https://nlp.stanford.edu/projects/snli/): a large-scale dataset for general-domain NLI.
- MedNLI (https://physionet.org/content/mednli/): a clinical-domain dataset annotated by medical experts, used to test domain generalization.

Data Preprocessing:

The data preprocessing pipeline applied to both datasets involved several key steps to ensure data consistency and facilitate effective sentence embedding generation. Initially, the datasets were loaded using either direct file extraction methods (e.g., '.xlsx', '.json') or through the Hugging Face dataset loader. Each sentence pair was cleaned by trimming extra spaces and formatted as tuples of the form (sentence 1, sentence 2), resulting in a list of sentence pairs ready for processing.

Tokenization was conducted using the pre-trained tokenizers associated with each transformer model (BERT, GPT-3, RoBERTa, XLNet), preserving punctuation as it may carry semantic significance in NLI tasks. Text was also converted to lowercase to maintain uniformity across datasets. Following the recommendation in a previous study (cited in the paper), stopword removal was not applied, as certain stopwords can play a contextual role in identifying sentence relationships. Additionally, the Hugging Face API was leveraged to simplify the process of loading pre-trained model weights and tokenizers for both PyTorch and TensorFlow frameworks.

Assessment Metrics:

Model performance was evaluated using accuracy for binary classification and both accuracy and macro-averaged F1-score in the updated three-way classification setting. The macro-F1 score was adopted to better capture class-wise performance when distinguishing between entailment, contradiction, and neutral cases. These metrics allow us to assess the geometric method's behavior in both simplified and fully realistic NLI scenarios.

4. Conclusion

Limitations:

Despite the competitive performance demonstrated on both SNLI and MedNLI datasets, the proposed geometric comparison method is not without limitations. The reliance on pre-trained transformer embeddings without fine-tuning may restrict its effectiveness in domain-specific NLI tasks, although the initial results with MedNLI suggest some promising potential. Additionally, the use of simple norm-based comparisons may not fully capture intricate semantic relationships, particularly in complex contradictions or multi-sentence inferences. Future work could address these limitations by integrating external syntactic or semantic features, incorporating fine-tuning with domain-specific data, and expanding the evaluation to more diverse NLI datasets.
